# Highly sensitive early-onset Alzheimer's disease: a case report

**DOI:** 10.3389/fpsyg.2025.1688924

**Published:** 2025-11-27

**Authors:** José Ángel Rubiño-Díaz, Melania Zapata-Moreno

**Affiliations:** 1 Functional and Clinical Neurophysiology: Biological Rhythms and Language (NRL) Group, Department of Biology, University of the Balearic Islands (UIB), Palma, Spain; 2 Health Research Institute of the Balearic Islands (IdISBa), Palma, Spain; 3 Son Espases University Hospital (HUSE), Palma, Spain; 4 Neuropsychology and Cognition Research Group, Department of Psychology, University of the Balearic Islands (UIB), Palma, Spain; 5 University Institute for Research in Health Sciences (IUNICS), University of the Balearic Islands (UIB), Palma, Spain; 6 University College Alberta Giménez-Comillas Pontifical University (CESAG-UP Comillas), Palma, Spain; 7 RAICES ZAPATA, Specialized Centre in Neuropsychology and Psychology, Palma, Spain

**Keywords:** sensory processing sensitivity, highly sensitive person, early-onset Alzheimer's disease, temperament trait, personality, neuropsychology, holistic and integrative approach

## Abstract

**Background:**

Early-onset Alzheimer's disease (EOAD) is an atypical syndrome that can be confused with other neurodegenerative diseases. This disease presents before the age of 65, with symptoms that generally affect executive functions, praxis, and visuoperceptual abilities, as opposed to episodic memory. Highly sensitive individuals present the temperament trait of sensory processing sensitivity, which is characterized by a differential susceptibility compared to other individuals. Neuropsychological evaluation should involve a holistic and integrative person-centered care approach for optimal treatment and disease progression.

**Case summary:**

A highly sensitive 54-year-old individual was diagnosed with EOAD at age 47 in 2017. Neuropsychological follow-up was conducted for 6 years. Initial neuropsychological testing revealed a cognitive pattern with impairments in executive functions, attention, and visual perception, the advancement of which led to a progressive deterioration in daily, occupational, and social functioning. During this period, he received psychotherapy from a psychologist specializing in neuropsychology and high sensitivity, using a holistic and integrative approach. Initially, sessions were held twice a week throughout the first year of consultation and, subsequently, continued at the patient's home and in his usual context, using a completely ecological perspective and consisting of person-centered care. In 2022, the patient, aged 59, was admitted to a nursing home. This situation, outside his usual environment, without autobiographical references and his own life story, led to accelerated deterioration, with the patient ultimately dying at age 60, in 2023.

**Conclusion:**

The patient with highly sensitive EOAD was followed for 6 years by a psychologist specializing in neuropsychology and high sensitivity. Neuropsychological intervention was maintained with a holistic and integrative person-centered approach using the unmet needs model to address cognitive, psychological, and functional levels. Follow-up with this approach could be key to slowing the disease and ensuring patient satisfaction throughout the entire progression of the illness. Greater visibility into unusual cases like this will enable psychology professionals to be vigilant for timely differential and diagnostic testing, which will significantly impact the treatment and progression of the illness, ultimately influencing quality of life and well-being through an optimal neuropsychological approach.

## Introduction

The cognitive, psychological, and functional changes of aging fall along a continuum ranging from a cognitively healthy state to dementia (Contador et al., 2024; Jessen et al., 2014). Subjective cognitive decline (SCD) has been defined as an increase in cognitive risk characterized by subjective cognitive complaints that cannot be explained by other health conditions, with no evidence of objective cognitive or functional impairment (Jessen et al., 2014); and mild cognitive impairment (MCI; Petersen, 2016) is considered one of the main threats in older adults (Bishop et al., 2010). Although different factors play a role, age is a determining, non-modifiable factor, (Fages-Masmiquel et al., 2021; Garre-Olmo, 2018). Neuropathological changes begin at least a decade before the clinical manifestations of the disease (Jack et al., 2013; Nelson et al., 2009; Price and Morris, 1999; Weiner et al., 2013). The prevalence of cognitive impairment in Spain is 18.5% in women and 14.3% in men; while among older adults over 80 years of age, prevalence reaches 45.3% (Vega et al., 2018; Vega-Quintana et al., 2018). Existing data on the conversion rate to Alzheimer's disease (AD) in patients with MCI vary widely from one study to another. According to Cardinali et al. (2010), the rate is generally 12% per year, although progression varies according to the MCI subtype (Palmer et al., 2008); whereas other studies estimate the percentage between 4% and 25% (Bozoki et al., 2001; Burns and Zaudig, 2002).

Alzheimer's disease is the most common form of dementia, accounting for between 50% and 75% of dementia cases (Alzheimer's Association, 2019). The two main categories are early-onset AD (EOAD) and late-onset AD (LOAD), based on an arbitrary criterion established by age 65 (Barber et al., 2017; Nudelman et al., 2023). EOAD affects people under 65 years of age and accounts for approximately 5% to 10% of AD cases (Dementia, 2006). A meta-analysis on EOAD showed that age-standardized incidence rates increase from 0.17/100,000 person-years in the 30–34 age group to 5.14/100,000 person-years in the 60–64 age group, resulting in an overall incidence rate of 11/100,000 person-years in the 30–64 age group. On average, incidence rates double every 5 years in the 40-year-old age group, which corresponds to an annual incidence of 370,000 new cases per year (Hendriks et al., 2023).

EOAD can result from genetic mutations (APP, PSEN1, PSEN2, GRN, and MPT), which can be inherited among family members (Dai et al., 2018; Giau et al., 2019). This represents a subset of EOAD cases, although it is possible to develop early-onset Alzheimer's disease due to causes other than genetic ones (Bateman et al., 2011). It is unclear to what extent the neuropathology underlying EOAD differs from that of LOAD (Palasí et al., 2015; Tellechea et al., 2018), but some distinctions have been reported. Typically, beta-amyloid (Aβ) neuritic plaque burden and intraneural deposition of neurofibrillary protein tangles (tau) involve brain regions in a characteristic pattern: Aβ initially accumulates in neocortical regions, then in the limbic system, diencephalon, basal forebrain, and finally in the cerebellum. Tau protein, on the other hand, first affects the entorhinal cortex and hippocampus, causing neurofibrillary degeneration, synaptic and neuronal loss, and regional atrophy in the early stages. Later (early/middle stages), the locus coeruleus, basal forebrain, and associated regions of the neocortex are affected, followed by the primary sensory cortex. Thus, both categories of neocortex, particularly the parietal and occipitoparietal regions, appear to experience a similar load, whereas the hippocampus is more likely to be unaffected in EOAD (Graff-Radford et al., 2021; Whitwell et al., 2012). Notably, involvement of the locus coeruleus is significantly implicated in the psychological and behavioral disturbances encountered in EOAD (Veréb et al., 2023) and involvement of the prefrontal cortex has implications for adaptive cognitive behaviors (Hanganu-Opatz et al., 2023). Individuals who develop AD at a younger age often appear to have a “purer” AD pathology with fewer concomitant neuropathological changes than older patients, who often display a constellation of associated pathologies (Boyle et al., 2019). This neuropathological condition causes affected individuals to experience cognitive symptoms with an atypical presentation: preserved episodic memory, but with impairments in other functions such as language, visuospatial ability, praxis, executive functions, and calculation. Changes in personality and behavior are initially subtle, such as irritation, apathy, and lack of motivation for activities they previously enjoyed (Alzheimer's Association, 2019; van der Lee et al., 2018).

Currently, an important aspect that remains unexplored is the cognitive functioning of older adults with the highly sensitive person trait (also known as sensitive person, HSP, or sensory processing sensitivity, SPS). HSPs do not have a psychological disorder or a disease. It is a personality trait, a temperament trait, that is present in 20% of the population; it is not something you have, it is something you are (Aron and Aron, 1997; Aron et al., 2010). There are no precise data in the older adult population.

People with SPS have a nervous system that perceives and processes more sensory information simultaneously and more deeply than usual. Although no differences have been found regarding brain structures or sensory organs, studies using functional neuroimaging tests have observed greater activation in areas of the premotor cortex related to the “wait and see” strategy in people with higher scores on the high sensitivity scale. Furthermore, greater activation of the medial temporal gyrus and the insula, involved in detecting another's mood and empathy, have also been observed. Moreover, the inferior frontal gyrus, a center associated with mirror neurons, has been seen to show greater activation for positive emotions (Acevedo et al., 2014, 2018).

Along these lines, people with SPS are exposed to a greater number of stressors, are more responsive to their environment, and often experience feelings of being overwhelmed, thereby conferring differential susceptibility in HSPs (Jagiellowicz et al., 2016). Aron and Aron (1997) also suggested that individual differences in SPS are partially determined by the reactivity of the behavioral inhibition system (BIS), which, along with the behavioral activation system (BAS) and the fight/flight system, is one of the three brain systems that appear to control emotional behavior (Gray, 1991). Generally, individuals with SPS exhibit heightened sensory awareness, both physical (e.g., sounds, smells, emotional expressions of others; Aron et al., 2012; Jagiellowicz et al., 2016) and psychological (e.g., increased empathy, depth of processing, self-reflection; Acevedo et al., 2018; Aron and Aron, 1997), and not limited to the individual (Acevedo et al., 2017; Aron et al., 2012; Jagiellowicz et al., 2011). Additionally, people with the SPS trait process sensory information more deeply, resulting in more intense emotional reactivity, heightened awareness of subtle stimuli, and a lower threshold for stimulation. Therefore, they tend to be overstimulated (Acevedo et al., 2017; Jagiellowicz et al., 2011; Yano et al., 2021, 2020). In this context of scientific literature, the analysis of this case enables a first approximation to EOAD with the SPS trait. The description of this case in its evolutionary process reflects the clinical manifestations at the cognitive, psychological, behavioral, and functional levels associated with the SPS trait. Another important aspect to consider is the premorbid personality of older adults with the SPS trait. Currently, it is not considered by neuropsychology professionals in neuropsychological evaluation and treatment, probably due to a general lack of awareness. Therefore, these individuals present a differential susceptibility that goes unnoticed. The neuropsychological approach to older adults with SPS should be holistic (Wilson et al., 2020) and based on the person-centered care model (PCC; Díaz-Veiga et al., 2015). In this sense, implications in evaluation and treatment are required to prevent or reduce cognitive, psychological, behavioral, and functional alterations at different stages of the process in people with a major neurocognitive disorder.

It is important to understand that neuropsychological rehabilitation has different approaches, including holistic neuropsychological rehabilitation. Wilson et al. (2020) explain that neuropsychological rehabilitation should focus on goals related to the patient's daily life and should be implemented wherever the patient lives. Only if this is not possible, should we ensure that a skill similar to that environment is trained. Furthermore, the person-centered care (PCC) model is a professionalized model that seeks to provide care by supporting people in maintaining control over their environment, their care, and their daily lives, developing their abilities, and feeling good. It seeks to improve the quality of care from dimensions related to quality of life. It provides a vision that starts from the recognition of the value and uniqueness of the person, focuses on abilities (vs. deficits), and supports the self-determination of individuals. Within the PCC model, we find the contributions of Tom Kitwood on the unmet needs of people with dementia, he says, “the lack of understanding of the needs of people with dementia and a negative and unsympathetic interaction with them, could be the cause of many behavioral disorders, which are nothing more than the way in which people with dementia express their discomfort and their unmet psychological, physiological and social needs” (Kitwood and Brooker, 2019; Díaz-Veiga et al., 2015).

The presentation of this case is based on the hypothesis that individuals with EOAD and the SPS trait may constitute a modifiable risk factor in the progression of the disease. A neuropsychological approach to these individuals' assessment, treatment, and follow-up by a psychologist specializing in clinical neuropsychology and high sensitivity, using a holistic and integrative person-centered approach, will help slow the progression of the disease and improve quality of life and well-being.

The objective of this study was to evaluate a case of highly sensitive early-onset Alzheimer's disease undergoing neuropsychological follow-up over 6 years.

## Case description and diagnostic guidance

A 51-year-old patient diagnosed with Early-Onset Alzheimer's Disease in 2015 at the neurology clinic, at the age of 47. But the first non-clinical symptoms appeared 2 or 3 years before 2011, at the age of 47. Several years later, in September 2017, the 54-year-old patient attended a neuropsychology clinic accompanied by his son, where an assessment and neuropsychological intervention. The patient presented with major neurocognitive disorder due to probable Alzheimer's disease according to DSM-5 criteria, a significant decline in one or more cognitive functions (such as memory, attention, language, executive function) that interfered with independence in daily activities. Furthermore, the patient was not in a state of delirium, nor was the decline attributed to another mental disorder.

The patient had no known drug allergies and no toxic habits. He had rhinitis and allergic asthma with positive tests for olive and pine pollen and dust mites. He had periods of sick leave due to stress. In 2011, he began seeing a neurologist for frequent memory lapses since May. At this first consultation, cognitive impairment associated with obstructive sleep apnea vs. pseudodeterioration was suspected, and tests were requested. Since 2020, he had grade 1 obesity and dyslipidaemia, for which he was undergoing dietary treatment. In June 2020, he had a possible episode of tonic-clonic seizure. His surgical history included nasal polyps and phimosis. There was no family medical history of cognitive impairment or Alzheimer's disease. The usual pharmacological treatment and treatment regimen are described below: donepezil: 10 mg (0-0-1), memantine 10 mg (1-0-0), flixonase 50 mcg/spray, ebastine 10 mg if required, salbutamol 100 mcg/inhalation (2 inhalations if necessary), levetiracetam 1000 mg (1-0-1) with reduction to 750 mg (1-0-1) in July 2021, and initiation of quetiapine 50 mg (0-0-1) on a progressive schedule in February 2022.

He was a social worker, head of department, and founder of social services in a town in Mallorca. He had multiple interests in his free time, devoting himself to hiking, composting, and beekeeping, and also volunteered in maritime rescue. Separated and the father of three children, he did not have a good relationship with his mother or his two brothers. Although he lived with his mother at the onset of the disease, after starting a relationship, he changed his address from 2017 to 2022.

In 2011, at the age of 47, he had begun experiencing recent lapses of memory, which lasted 2 to 3 years. He needed to write everything down, and made greater use of diaries, but this had little impact on his work. In addition, the patient presented subjective disorientation and work-related stress. The diagnostic approach (DA) was cognitive impairment associated with obstructive sleep apnea/hypopnea syndrome (OSAHS) vs. pseudo-impairment.

In 2012, a non-contrast cranial magnetic resonance imaging (MRI) was performed, the findings of which ruled out significant alterations in the brain parenchyma.

In October 2015, at 52 years of age, the radiological study was repeated, finding no significant alterations. The patient was referred for a neuropsychological evaluation that same month. The examination concluded diffuse cognitive impairment affecting processing speed, simple and complex attentional processes, executive functions, visuoconstructive functions, and memory. At the functional level, the patient began experiencing difficulties with advanced activities of daily living. Therefore, the neuropsychological evaluation was decisive and provided information to establish the diagnostic orientation of Alzheimer's disease.

In 2016, the patient was 53 years old and underwent a positron emission tomography (PET)-computed tomography (CT) scan of the brain, which revealed indirect signs of atrophy predominantly in the anterior fronto-medial region. Marked bilateral decreased uptake of the parietal and posterior temporal cortex with extension to the lateral occipital cortex was also observed. The frontal cortex was mildly hypometabolic, but basal ganglia were preserved. This concluded a pattern of Alzheimer's disease (AD).

In 2017, at age 54, a follow-up neuropsychological evaluation was performed, and neuropsychological intervention was initiated for the patient with mild dementia due to AD (DG). At the functional level, 19% of activities of daily living were performed under supervision or with assistance. The cognitive profile showed impairment in fixation and delayed recall, impairment of visuoperceptual and visuoconstructive functions, visual agnosia, constructional and ideomotor apraxia, bradypsychia, and impairment of executive functions.

In 2018, the patient was 55 years old, and a follow-up neuropsychological evaluation was requested by a forensic clinical psychologist for the processing of legal incapacity. The results were as follows: Mini-Cognitive Examination (MCE): 22/35; Mini-Mental State Examination (MMSE): 24/30; Montreal Cognitive Assessment (MoCA): 13/30; Goldberg Anxiety and Depression Scale: no symptoms; Activities of Daily Living (ADL) with the Barthel Index: 100/100; and Instrumental Activities of Daily Living (IADL) with the Lawton and Brody Index: 7/8. He also presented mild temporal disorientation, remaining oriented in space and person, with a good capacity for sustained attention, but slowed selective attention under high stimulus load. He also presented difficulties with immediate visual/verbal memory and recalling information; moderate impairment in working memory, but with active listening, he easily resumed his own conversation. He also displayed mild anomias and circumlocutions during speech, and mild anomias in naming by visual confrontation. His reading and writing skills were preserved, although he made some errors. He retained number recognition, but with difficulty calculating. He also presented moderate visuospatial/visuoperceptual/visuoconstructive difficulties, and mild impairment in complex visual planning and sequencing processes. He exhibited good abstract verbal reasoning skills and verbal fluency with phonetic cues. Finally, it should be noted that there was no deficit in response inhibition; he maintained good judgment and decision-making skills, albeit with a slowdown in information processing.

In 2019, the patient was 56 years old and underwent a follow-up evaluation in the neurology department. He reported well-established routines, walking independently without disorientation, independent activities of daily living (only supervision in clothing selection), but difficulties with instrumental activities such as handling money and making large purchases. At that time, he was awaiting legal incapacity. The conclusion was a DG: AD in the moderate or moderately severe phase.

In July 2020, the neurology department reported an epileptic seizure in a patient with AD: a generalized seizure while on vacation away from his usual place of residence (June 2020): with tongue biting and sphincter relaxation. MRI revealed an increase in the depth of the subarachnoid space sulci and fissures, proportional to moderate dilatation of the ventricular system, associated with cortico-subcortical atrophy disproportionate to the patient's age. Levetiracetam 1000 mg was administered every 12 h.

In September 2020, a change occurred during the neuropsychological intervention sessions. After months of confinement and the epileptic seizure, the patient's cognitive and functional status worsened. During this period, the number of cognitive stimulation sessions was reduced.

In July 2021, at the age of 58, the neurology department reported a slow deterioration (more aphasic, more apraxic, spatially disoriented, loss of IADLs, and dependence in some BADLs (dressing, hygiene, and toileting). He presented with moderately severe Alzheimer's disease. The possible episode of tonic-clonic seizure was well controlled and there were no behavioral disorders.

In April 2021, based on observations during the intervention sessions and data collected in discussions with his family, his cognitive profile had evolved significantly over the previous year, albeit slowly and gradually. This was particularly true at the functional level. As of January 2021, he had required 24-hour supervision because he was unable to take his medication independently, had difficulty dressing and with personal hygiene, and also with toileting. Cognitively, he had begun to become spatially disoriented and had stopped going out alone for walks. In addition, he had difficulty sequencing steps, and moderate anomia, which meant he had difficulty understanding his speech, his working memory was more affected, and he was beginning to experience difficulties with his autobiographical memory. During this period, he experienced no emotional or behavioral disturbances, remaining aware of his illness, expressing his difficulties and the need to continue working to slow the process.

In June 2021, he requested occupational therapy but was unsuccessful. In February 2022, at age 59, he underwent neuropsychological follow-up. The results of the tests used were as follows: MMSE: 8/30; Barthel index: 60/100; Lawton and Brody index: 1/10; and a decline in spatial and temporal orientation, visuoperceptual/visuospatial functions, and praxic functions. During this period, he was no longer able to read or write, although he did try to read, making numerous errors and not placing words in the correct order. He had moderate anomia, but attempted to express himself so that he could be understood. The decline in his working memory made it difficult to redirect conversations. During this period, he no longer remembered events from his autobiography and presented agitation that increased in the evening with sleep disturbances. In February 2022, his relationship broke down and he moved out of his home; therefore, he returned to live with his mother. He began 24-hour care. Behavioral disorders appeared. In August 2022, he was admitted to a nursing home, and in May 2023, the patient died.

## Sensory processing sensitivity

In November 2020, Dr. Elaine Aron administered the basic test for the trait of Sensory Processing Sensitivity (SPS; Aron and Aron, 1997). This very simple test consists of 23 dichotomous true-false questions that gather information about heightened emotional sensitivity and reactivity (reaction) to internal and external stimuli because the nervous system of an HSP produces heightened awareness and responsiveness to environmental, social, physical, and sensory stimuli. The patient answered “yes” or “no,” and the number of times they answered “yes” was counted. The result showed 23 points out of a total of 23, indicating that the patient was a highly sensitive person. Following this test, the trait was taken into account to better tailor the intervention to the patient's needs. The most notable characteristics related to the four elements of SPS in terms of the case are described below.

### Deep cognitive processing of information

He had a rich and complex inner life; he was a very introspective person with multiple interests. During his illness, he maintained his capacity for insight, which enabled him to express his emotions and thoughts about his illness and its progress. During the intervention, he was also able to request the things he felt he needed to maintain his autonomy for as long as possible and to suggest topics of interest to create therapeutic content. Loss of autonomy over time led him to deeply reflect on his own role. By the end of the illness, it was difficult to appreciate this element due to the severity of his cognitive function.

### High emotionality and empathy

He was deeply moved by all information related to art. Music was one of his greatest sources of connection and inspiration. During many therapeutic moments, it was necessary to help him regulate intense emotional states that were caused by the fact that his family's behavior and their treatment of his illness greatly affected him. He experienced emotional instability from feeling misunderstood and not allowed to be the protagonist of his life. Despite the progression of his illness, he was usually attentive to what he could do to help and how; always willing to lessen the burden on those around him. He was very involved in issues of social injustice, the environment, ecology, volunteering, and helping disadvantaged people and broken families.

### Sensory sensitivity and sensitivity to subtleties

The patient was very aware of the subtleties of his environment and was able to recognize what other people needed. For example, during his therapist's pregnancy, he would walk ahead of them on hiking trails and alert her to rocks and places where she should be careful while walking. He was bothered by loud noises, certain rough clothing fabrics, and also strong odors.

### Ease of overstimulation

Due to his differential susceptibility, the patient required a lot of time spent alone in order to maintain his own balance, needing many spaces to connect with nature. Throughout his life, he tended to seek solitude in nature to find his own inner balance. During the cognitive assessments he underwent in the hospital, he was aware that he would freeze, feel pressured, and often reported that people were talking in front of him as if he weren't there. He felt that the performance he displayed during these appointments wasn't what he was capable of in his daily life.

At the beginning of the illness, all the factors were relevant, and as such were taken into account so as to accommodate and manage his daily life according to his internal and external needs. As the illness progressed, starting in February 2022, the factors of sensory sensitivity and the tendency to overstimulation were prioritized, so that the environment would be as sensitive as possible to his sensory needs and he could be more comfortable and calmer.

**Figure 1:**
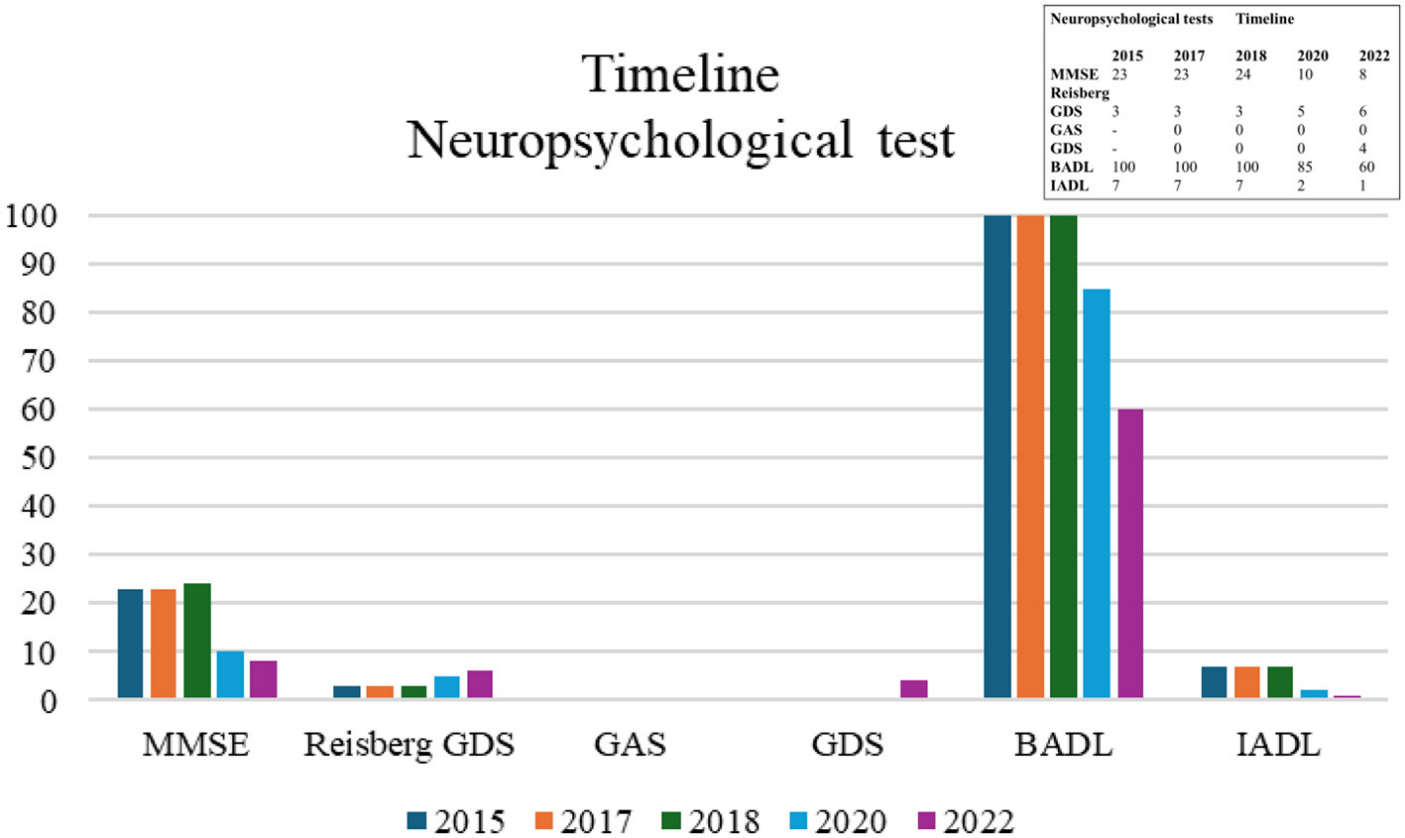
Timeline of direct scores from neuropsychological tests administered from 2015 to 2022. BADL, Basic Activities of Daily Living (cut-off score < 100 is pathological; González et al., 2018); GAS, Goldberg Anxiety Scale (cut-off score ≥ 2; Montón-Franco et al., 1993); GDS, Goldberg Depression Scale (cut-off score ≥ 1; Montón-Franco et al., 1993); IADL, Instrumental Activities of Daily Living (cut-off score < 8 is pathological; Vergara et al., 2012); MMSE, Mini Mental State Examination (cut-off score 26/30, Blesa et al., 2001); Reisberg GDS, Reisberg Global Deterioration Scale (cut-off score ≥2 is pathological, Blesa et al., 2001).

Figure 1 presents a timeline of clinical evolution at the cognitive, functional, and mood levels from 2017 to 2022. The evolutionary decline is shown through direct scores with the neuropsychological tests used. The Mini Mental State Examination (MMSE, Folstein et al., 1975) in 2015 was 23 points and also 23 points in 2017. In 2018 it reached 24 points, followed by 10 points in 2019 where there was a significant decline, finally dropping to 8 points in 2022. In addition, the Global Deterioration Scale (GDS, Reisberg et al., 1986) was used. This corresponded, from 2015 to 2018, to a GDS score of 3, indicating mild cognitive impairment compatible with incipient Alzheimer's disease. In 2020, a GDS score of 5 indicated moderately severe cognitive impairment consistent with moderate dementia; and, finally, in 2022, a GDS score of 6 indicated severe cognitive impairment consistent with moderately severe dementia. On the Goldberg Anxiety and Depression Scale (GHQ-18, Goldberg, 1972; Goldberg and Hillier, 1979; Godoy-Izquierdo et al., 2002), the anxiety component was scored zero each year the assessment was conducted. This indicated that he did not experience overt anxiety that affected his daily life. On the depression component, he scored 4 in 2022, indicating symptoms of mild depression. In activities of daily living (ADL, Mahoney and Barthel, 1965), he presented a score of 100 from 2015 to 2018, which indicated an independent person for ADL, whereas in 2020 he presented a score of 85, and 60 points in 2022. This indicated a mild degree of dependence. In instrumental activities of daily living (ADL, Lawton and Brody, 1969), he presented a score of 7 from 2015 to 2018, then a score of 2 in 2020, and a score of 1 in 2022, indicating a worsening degree of dependence on a scale ranging between 0 points (maximum dependence) and 8 points (total independence).

## Intervention

A person-centered (holistic neuropsychological) intervention was carried out with an integrative holistic approach based on an ecological and unmet needs model. From 2017 to 2019, the patient was seen twice a week in consultation for 45 min. In January 2019, home care began once a week for 60 min and once in consultation. In October 2020, the patient received two home sessions per week and one in consultation. In 2022, the sessions were reduced to one 45-min home session per week and, starting in May, only 30 min. The intervention was completed at the end of June 2022. During this period, the neuropsychological approach was provided by a psychologist specializing in clinical neuropsychology, and high sensitivity.

Neuropsychological rehabilitation has different approaches, including holistic neuropsychological rehabilitation (Wilson et al., 2020) and the person-centered care model (PCC, Kitwood and Brooker, 2019; Díaz-Veiga et al., 2015).

From 2017 to 2019, the intervention focused on remembering her autobiography as much as possible, continuing to write down her thoughts, maintaining her functionality, helping her partner in any way she could, understanding the illness, and providing emotional support for her own grief and family issues. We began by developing a therapeutic bond by understanding her life story, collecting her tastes and interests, and preparing sufficiently motivating material. At the cognitive level, we worked on computer writing, where she shared her thoughts or meaningful activities she had done in the previous days. Through readings that captured her tastes and interests, we performed memory exercises using the space retrieval technique, decision-making, problem-solving, and writing. At the functional level, we worked with her partner to establish routines and habits regarding medication taking, telephone use, household chores, and self-care, respecting and taking into account her energy levels (physical, cognitive, and emotional). We also worked on orientation using maps of familiar areas to strengthen mental pathways to familiar places. At the same time, we introduced meaningful activities into his daily life, which we decided on during the consultation and shared with his partner (going to the movies every weekend, going out to dinner, attending classical music concerts, etc.). To maintain financial functioning, we worked on money management by presenting situations very similar to those he experienced in his daily life. As he found it more difficult to handle coins, the process was adapted to giving the closest bill to the amount requested, until finally he only carried a specific amount in his pocket. On an emotional level, in many sessions we needed to dedicate 10–15 min to talking about his family conflicts, and in this process, classical music helped him externalize his deepest emotions and release the discomfort he felt. At the family level, limits were established based on the patient's emotional needs (e.g., reducing visits from his mother). Starting in 2019, one of the sessions began at home. The sessions focused on making shopping lists and going to the supermarket, cooking, and going for walks along routes that were meaningful to him. At the same time, they discussed news of interest, family matters, and simply the routes. The routes provided sufficient stimulus to connect and explain aspects of his life story. Notes were taken from these sessions to later remind him of things he might no longer remember or to create his own autobiographical project. These types of activities were maintained until before the COVID-19 pandemic.

During the COVID-19 pandemic, along with the therapist's maternity leave, he stopped receiving intervention sessions with the therapist for almost 6 months. Therefore, in June and July, the intervention was replaced by another therapist.

Neuropsychological rehabilitation has different approaches, including holistic neuropsychological rehabilitation. Wilson et al. (2020) explain that neuropsychological rehabilitation should focus on goals related to the patient's daily life and should be implemented wherever the patient lives. Only if this is not possible should we ensure that a skill similar to that environment is trained.

From 2017 to 2020, the COVID-19 pandemic and the therapist's maternity leave resulted in the patient not receiving intervention sessions with the therapist for almost 6 months. In June and July, the intervention began to be taken over by another therapist.

From 2020 to 2021, the intervention focused on maintaining autonomy to be able to go for walks. We began by adding a GPS finder in 2020, and by 2021, a support person was included to maintain this daily routine. The oral expression of thoughts and emotions related to his grief, due to his heightened awareness of the illness, were facilitated. Listening comprehension was maintained through reading; naming and ideational praxis were worked on with objects from his daily life, critical thinking, and a PowerPoint presentation of his autobiography was worked on.

In 2022, the home intervention continued with the main objective of maintaining the bond with a support person until his new routine was stabilized again, as well as enabling his oral expression of thoughts and emotions. A routine with meaningful daily activities was reestablished through participation and observation and the narration of his autobiography was maintained. At the family level, in terms of care for the caregivers, on-demand family sessions were held to address and understand his needs and manage his behavioral disorders based on a model of unmet needs and person-centered care. The behavioral disorders appeared after the change of address, after returning to his mother's house, with whom he had never had a good emotional relationship. Informal caregivers were also trained to provide care using the same model and appropriately address the differential susceptibility he presented.

After completing the intervention with the patient, contact and follow-up were maintained with the son to provide support as needed in the moments and situations where it was required.

**Figure 2:**
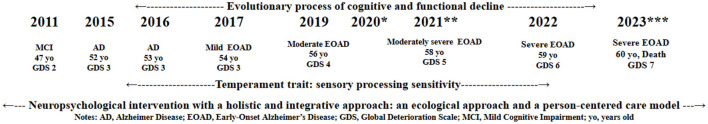
Progression of the patient with EOAD and the SPS temperament trait. AD, Alzheimer Disease; EOAD, Early Onset Alzheimer Disease; GDS, Global Deterioration Scale; MCI, Mild Cognitive Impairment; SPS, Sensory Processing Sensitivity; yo, years old. *March 2020, beginning of the COVID-19 pandemic period; **July 2021, possible episode of tonic-clonic seizure; ***May 2023, admission to a nursing home.

Figure 2 shows the timeline of the person-centered neuropsychological intervention with a humanistic integrative approach. From 2017 to 2022, the psychologist specializing in clinical neuropsychology and high sensitivity conducted a tailored intervention with the patient with EOAD and the SPS trait, as previously described.

## Discussion

The objective of this study was to evaluate a patient over 54 years of age with highly sensitive early-onset Alzheimer's disease (EOAD) under neuropsychological follow-up for 6 years.

A review of the patient's medical history shows that in 2011, at age 47, he was diagnosed with mild cognitive impairment, which remained stable in the cognitive and functional spheres until 2015, when he was diagnosed with mild EOAD. Difficulties with diagnosis in younger patients may mean that they are diagnosed later, making their progression appear more rapid. In this sense, research on the best screening methods will help improve early diagnosis (Ayodele et al., 2021). In 2017, at age 54, the patient was seen by a psychologist specializing in clinical neuropsychology and high sensitivity until 2022 when, at 59 years of age, he was admitted to a nursing home by family decision. The patient died in 2023, at the age of 60. This progression period showed a total duration of 14 years in a patient diagnosed with highly sensitive EOAD. Therefore, it is important to diagnose dementia early, as this will enable the provision of appropriate treatments, support, and resources, allowing for future planning (Balasa et al., 2011).

These data on the progression period of the disease contrast with the literature, as individuals with EOAD tend to show very rapid progression in cognitive and functional decline (Bateman et al., 2011). This case was diagnosed in 2011. The neuropsychological approach, based on person-centered care with a humanistic integrative focus, went on for 6 years, while the progression process lasted 14 years. This was inconsistent with previous research, which showed that people with EOAD have a median survival time after diagnosis of 7.9 years (Brodaty et al., 2012) although another recent study indicated an even longer survival time of over 10 years (Gerritsen et al., 2019). In this regard, it is important to note that the rapid deterioration that occurs in the patient when he was admitted to a nursing home. The patient was in a state of physical frailty and advanced cognitive impairment with executive dysfunction (Bartoli et al., 2020). This event could have created a social context that triggered traumatic and stressful emotional responses, such as social exclusion. In this sense, this patient with EOAD and SPS could be more vulnerable to social exclusion and social pain, which would explain their rapid physical and cognitive decline (Morellini et al., 2023). This implied a perception of social isolation (loneliness). From different perspectives, it is explained that loneliness has a negative impact on self-perception of health. Fundamentally, from a neuropsychological perspective, loneliness is a risk factor for poorer overall cognitive performance, faster cognitive decline, impaired executive functioning, and greater sensitivity to social threats (Morese and Palermo, 2022). Therefore, these data reflect the need to factor healthcare services into planning for people with EOAD. Further, it highlights the need for targeted interventions tailored to people with EOAD in order to slow the disease and improve quality of life throughout the course of the disease.

The progression of EOAD is difficult to estimate. While there is evidence that EOAD can progress more rapidly and aggressively (Bateman et al., 2011), experts are unsure whether this is conclusive. Furthermore, each person's experience, response to treatment, and progression with the disease are different (Sirkis et al., 2022).

The patient presented with EOAD with SPS. An important aspect worth noting is that this led to a sensitivity to the subtleties of the environment, meaning HSPs have a greater capacity to effectively process a greater number of sensory stimuli around them, yet information overload and stimulus overload can lead to mental saturation and sensory overstimulation. In this sense, this manifests as differential susceptibility (Aron et al., 2012; Greven et al., 2019; Lionetti et al., 2019), meaning HSPs are profoundly affected by their environments “for better or for worse.” This implies three investigated reasons why science is shifting its perception of SPS: (1) HSPs benefit more from psychological intervention for several reasons: their capacity for emotional healing is much greater—in this sense, HSPs can benefit from psychotherapy; they are more sensitive to positive stimuli, thus, HSPs can likely benefit from kind people in their environment and the positive effects of their life history; (2) HSPs adapt with a tendency to become invisible due to the need to disconnect from less stimulation, and are perceived for their creativity, level of awareness, efficiency, and endearment; and 3) When HSPs become aware of this trait, their social and romantic relationships improve.

Additionally, from a behavioral perspective, people with SPS are cautious, pausing and observing before acting (Lionetti et al., 2019). This appears to be due to a more active behavioral inhibition system (BIS), which assesses whether a stimulus should be approached or avoided. Moreover, this gives the body time to suppress behaviors that may lead to negative outcomes, or to avoid threatening or unrewarding stimuli (Amodio et al., 2008; McNaughton and Gray, 2000). This trait facilitates deeper, more precise cognitive processing of information and requires longer inhibition times in response to stimuli (Aron et al., 2012; Nachmias et al., 1996), but does not imply a lack of curiosity or aversion to new experiences.

The neuropsychological approach of person-centered care (Kitwood and Brooker, 2019; Díaz-Veiga et al., 2015) with a humanistic integrative focus (Wilson et al., 2020) involves structuring sessions in an ecological way, by adjusting to the temperament trait, thereby leading to greater control and possible delay of the disease. Furthermore, Ben-Yishay and Prigatano (1990), as references in the holistic or multifaceted theory that integrates rehabilitation and neuropsychology, also emphasize the importance of the interaction between cognitive, emotional, social, and functional alterations, and the need for a therapeutic alliance between patient, family, and professionals to improve the patient's quality of life. In this sense, they address the need to include the interests of patients to help them promote their rehabilitation. In this context, there is evidence that holistic neuropsychological rehabilitation is significantly effective for community reintegration, functional independence, and proactivity, in addition to improving attention, memory, social and communication skills, and executive functions, among others (Cicerone et al., 2011). Finally, in another study (Van Heugten et al., 2012), the same conclusions of efficacy were reached after a meta-analysis reviewing 95 randomized controlled trials between 1980 and 2010.

The approach to this patient with a psychologist with specialized training in the SPS temperament trait may have enhanced the therapeutic process and slowed progression. SPS is a biologically based trait (a temperament trait) associated with greater depth of processing, empathy and emotionality, awareness of subtleties, and responsiveness to stimuli (Acevedo et al., 2014, 2021; Acevedo, 2020; Aron and Aron, 1997; Pluess and Boniwell, 2015). In this sense, the neuropsychological approach must be individualized and tailored to the individual's specific characteristics.

People with early cognitive decline generally present amyloid and tau deposits in the cerebral cortex. This influences the default mode network (De Santi et al., 2023; Shirbandi et al., 2021). A highly sensitive person is suggested to be highly sensitive because scientific studies show that the default mode network presents greater neuronal activation, which does not occur in people without this trait. This would explain a possible heightened awareness of the disease, greater insight, and a possible more widespread progressive loss of this network.

## Limitations

This work has some limitations that should be addressed. First, the patient did not have genetic or cerebrospinal fluid testing available for personal reasons. Second, tau protein positron emission tomography (PET) and 18kDa translocator protein (TSPO) PET were not performed to demonstrate the multidimensional neuroimaging of AD. Third, this particular case of EOAD presented with the SPS trait, for which no solid evidence has been found in exposing the influence of this trait as a modifiable risk factor or protective factor for neurocognitive disorders in older adults. It is important to design future research that would allow for an in-depth, rigorous exploration of older adults with the SPS trait to answer various questions.

## Conclusions

This paper describes a case of highly sensitive early-onset Alzheimer's disease, followed by a psychologist specializing in neuropsychology and high sensitivity. Through observation and assessment during follow-up, the clinical manifestations of the cognitive, psychological, and functional spheres related to the highly sensitive person trait are provided in detail. Person-centered neuropsychological intervention is maintained with a holistic integrative approach based on an ecological and unmet needs model. Follow-up with this approach in highly sensitive individuals could be key to slowing the disease, and to the satisfaction and well-being of the individual in the developmental process. This approach aims to highlight individuals with the sensory processing sensitivity trait who are developing neurocognitive disorders, as it may be a modifiable risk factor and/or slow the progression of the disease when the approach is optimal, considering the particular characteristics of individuals with SPS. Greater visibility of unusual cases like this allows psychology professionals to be alert for a timely differential diagnosis and diagnosis, which will significantly impact the treatment and progression of the disease.

In the future, the presentation of this case will help change neuropsychological assessment and intervention protocols for person-centered care with a human-centered approach.

## Data Availability

The datasets presented in this article are not readily available because the data used are from neuropsychological testing assessments. These data did not require any additional processing. Requests to access the datasets should be directed to joseangel.rubino@uib.es; José Ángel Rubiño-Díaz.
